# Cloning of a gene-edited macaque monkey by somatic cell nuclear transfer

**DOI:** 10.1093/nsr/nwz003

**Published:** 2019-01-24

**Authors:** Zhen Liu, Yijun Cai, Zhaodi Liao, Yuting Xu, Yan Wang, Zhanyang Wang, Xiaoyu Jiang, Yuzhuo Li, Yong Lu, Yanhong Nie, Xiaotong Zhang, Chunyang Li, Xinyan Bian, Mu-ming Poo, Hung-Chun Chang, Qiang Sun

**Affiliations:** 1 Institute of Neuroscience, CAS Center for Excellence in Brain Science and Intelligence Technology, CAS Key Laboratory of Primate Neurobiology, State Key Laboratory of Neuroscience, Chinese Academy of Sciences, Shanghai 200031, China; 2 Shanghai Research Center for Brain Science and Brain-inspired Technology, Shanghai 200031, China

**Keywords:** macaque monkey cloning, somatic cell nuclear transfer, non-human primate models, CRISPR/Cas9 gene editing, circadian rhythm disorders

## Abstract

Cloning of macaque monkeys by somatic cell nucleus transfer (SCNT) allows the generation of monkeys with uniform genetic backgrounds that are useful for the development of non-human primate models of human diseases. Here, we report the feasibility of this approach by SCNT of fibroblasts from a macaque monkey (*Macaca fascicularis*), in which a core circadian transcription factor BMAL1 was knocked out by clustered regularly interspaced short palindromic repeat/Cas9 gene editing (see accompanying paper). Out of 325 SCNT embryos transferred into 65 surrogate monkeys, we cloned five macaque monkeys with *BMAL1* mutations in both alleles without mosaicism, with nuclear genes identical to that of the fibroblast donor monkey. Further peripheral blood mRNA analysis confirmed the complete absence of the wild-type *BMAL1* transcript. This study demonstrates that the SCNT approach could be used to generate cloned monkeys from fibroblasts of a young adult monkeys and paves the way for the development of macaque monkey disease models with uniform genetic backgrounds.

## INTRODUCTION

Non-human primates are useful animal models in biomedical research due to their proximity in evolution, and similarities in physiology and anatomy, to humans [1]. Transgenic monkeys carrying human disease-related genes can be generated by lentivirus-based introduction of human genes into monkey embryos [2–4]. With recent advances in gene-targeting technologies such as clustered regularly interspaced short palindromic repeat (CRISPR)/Cas9 editing, it has become feasible to generate monkey embryos and offspring with specific gene mutations or insertions at a relatively high efficiency [5–7]. However, transgenes introduced by viral vectors are inserted at random locations and with variable copies in the chromosomes [4]. Furthermore, mosaicism may occur in nuclease-based gene editing of the embryo [5,8–10], whereby multiple genotypes are created in different tissue cells of a single animal, which complicates phenotypic analysis. Cross-breeding of founders has been the common solution for eliminating mosaicism in mice, but is unpractical for monkeys because of the long duration of the generation cycle [11,12]. Acceleration of sperm maturation by xenografting testis tissues could reduce the cycle time by about one-half [13], but oocyte maturation remains slow. Finally, monkeys used for gene editing so far have come from diverse genetic backgrounds, imposing serious challenges in their use as animal models in pre-clinical studies of therapeutic treatments in a manner similar to mouse disease models.

Somatic cell nuclear transfer (SCNT) using gene-edited donor somatic cells can generate a group of genetically uniform gene-modified animals without mosaicism and cross-breeding. Thus, the SCNT-based approach is particularly valuable for generating gene-modified monkey models [14]. We have recently demonstrated the feasibility of monkey SCNT and obtained two monkeys using fetal fibroblasts as somatic cell donors [15]. In the present study, we further developed the monkey SCNT approach by generating five monkey offspring using fibroblasts from a young adult macaque monkey. Moreover, the latter ‘founder' monkey was genetically modified by CRISPR/Cas9 editing of a core circadian gene, *Brain and Muscle ARNT-Like 1* (*BMAL1*), in zygotes (see accompanying paper). Thus, monkey cloning by SCNT could be used to generate non-human primate models of gene-based human diseases.

## RESULTS

### Preparation of somatic cells from a BMAL1 knockout monkey

**Figure 1.**
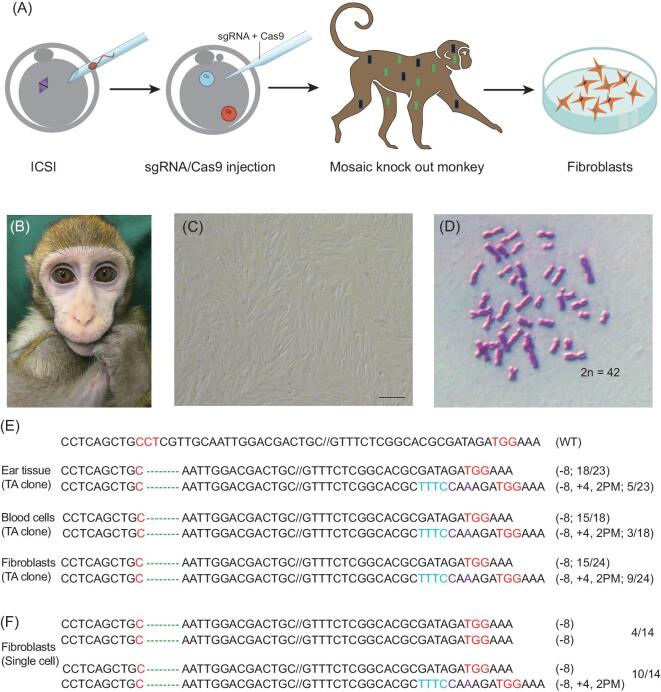
Preparation of fibroblasts from a *BMAL1*-edited monkey. (A) A schematic diagram depicting the procedure of culturing fibroblasts. (B) *BMAL1*-edited founder monkey A6 (see accompanying paper). (C) Primary fibroblast culture derived from the skin of the A6 founder monkey; scale bar, 200 μm. (D) Normal karyotype of A6 fibroblasts. (E) TA clone analysis of the PCR products using DNA (ear tissue, blood cell and fibroblasts) from the A6 monkey. (F) Single-cell genotype analysis of A6 fibroblasts.

BMAL1 is a core transcription factor involved in regulating circadian rhythm [16,17]. In the accompanying paper, we have reported the generation of five *BMAL1* gene-edited monkeys by zygote editing via the CRISPR/Cas9 method. Among the five monkeys, we chose monkey A6 as the founder monkey for cloning because this monkey exhibited a complete absence of the *BMAL1* transcript and strong circadian disorder phenotypes, including dampened circadian cycling of blood hormones, elevated nocturnal locomotive activity, reduced rapid eye movement (REM) and non-REM sleep, as well as psychosis-related behaviors. Skin fibroblasts were obtained from monkey A6 and cultured prior to their use for SCNT (Fig. 1A–C).

Karyotyping of cultured fibroblasts showed a normal diploid set of 42 chromosomes (Fig. 1D). Genotype analysis by sequencing the TA clones of polymerase chain reaction (PCR) products that were amplified using DNA from the ear tissue, blood cells and fibroblasts of the A6 founder monkey revealed two types of *BMAL1* mutation: an 8-bp deletion (‘−8') and an 8-bp deletion together with a 4-bp insertion and 2-bp point mutation (‘−8, +4,2PM') (Fig. 1E). Further genotype analysis of single fibroblast cells showed either a homogeneous bi-allelic mutation of ‘−8/−8' or heterogeneous bi-allelic mutation of ‘−8/−8, +4,2PM' in the *BMAL1* gene (Fig. 1F). As shown in the accompanying paper, *BMAL1* editing resulted in the same two *BMAL1* genotype, and the complete absence of *BMAL1* expression in the founder monkey A6.

### SCNT using fibroblasts of the *BMAL1*-edited monkey

**Figure 2.**
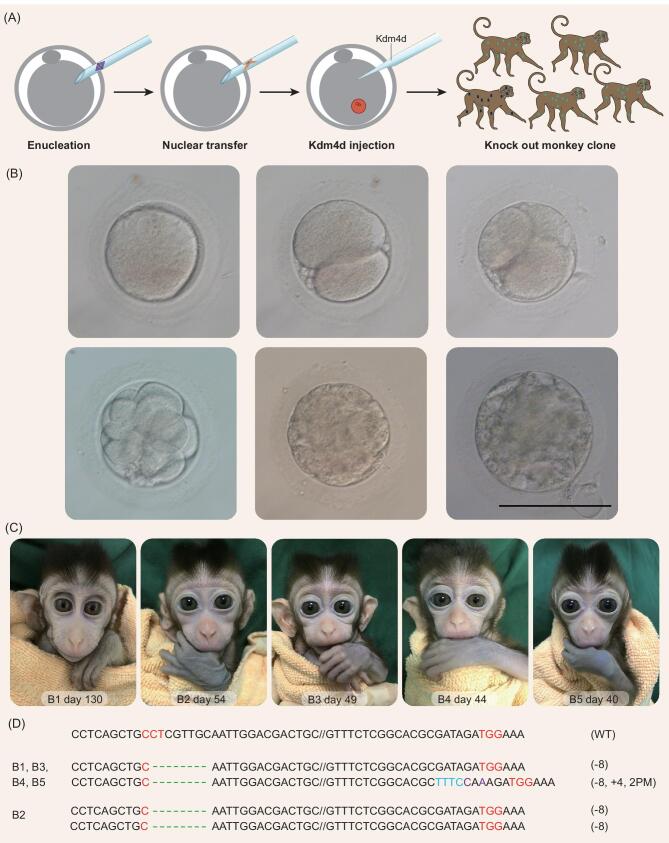
Generation of monkey offspring by SCNT using fibroblasts from a BMAL1 knockout monkey. (A) A schematic diagram depicting the procedure of generating *BMAL1* knockout monkey clones. (B) Example images of monkey SCNT embryos at different stages; scale bar, 120 μm. (C) Images of B1–B5, five cloned cynomolgus monkeys generated by SCNT using fibroblasts from the BMAL1 knockout monkey A6. (D) BMAL1 mutation analysis on ear tissue from all five cloned monkeys, showing that four monkeys carry heterogeneous bi-allelic (−8/−8, +4, 2PM) and one carries homogeneous bi-allelic (−8/−8) mutation of the *BMAL1* gene.

Using the same protocol described in the previous study [15], we performed SCNT using fibroblasts derived from *BMAL1*-edited macaque monkey A6. As all of the detected donor cells carried mutations leading to the loss of function of the *BMAL1* gene, we did not further purify different cell types among the clonal lines of fibroblasts. Mature oocytes were obtained by super-ovulation of female macaque monkeys [18]. Reconstructed SCNT oocytes were obtained by Sendi virus-assisted fusion of single fibroblasts with enucleated oocytes, and further activated by incubation with ionomycin and 6-dimethylaminopurine. To facilitate the epigenetic reprogramming of the somatic nucleus, one-cell-stage SCNT embryos were incubated with a histone deacetylase inhibitor trichostatin A (TSA) and injected with mRNA expressing H3K9me3 demethylase Kdm4d, as described previously [15] (Fig. 2A, B). Similar to that reported for SCNT embryos derived from fetal fibroblasts [15], we found a high percentage of blastocyst formation (10/17, 58.8%) for SCNT embryos using fibroblasts obtained from the *BMAL1*-edited monkey A6. Among these blastocysts, 80% (8/10) formed a prominent inner cell mass (ICM), a sign for normal embryo development. Given the high efficiency of blastocyst and ICM formation, we transferred 325 SCNT embryos at the early (two-to-eight-cell) blastomere stages to 65 surrogate female monkeys, and found 16 pregnancies (Supplementary Table S1). Five live births (B1–B5) were obtained, all of which survived well under human-assisted feeding (51–141 d at the time of manuscript submission) (Fig. 2C, Table 1). As shown in Table 1, the best pregnancy and live-birth rates were found for fibroblasts that underwent four passages.

**Table 1.** Statistics for the development of the SCNT embryos.

Cell passage	Embryos transferred	Surrogates	Pregnancies	Live birth (Number)
2	118	23	7	1 (B1)
3	148	30	4	1 (B2)
4	59	12	5	3 (B3, B4 and B5)
Total	325	65	16	5

### Genetic analysis of cloned monkeys

We first analyzed the *BMAL1* genotype of the five cloned monkeys using the ear tissues. We found that four cloned monkeys (B1, B3, B4 and B5) carried the heterogeneous bi-allelic mutation (−8/−8, +4,2PM) and one monkey (B2) carried homogeneous bi-allelic mutation (−8/−8) of the *BMAL1* gene (Fig. 2D). This is identical to the mutation genotypes of donor fibroblasts described above and those of the founder monkey A6.

**Figure 3.**
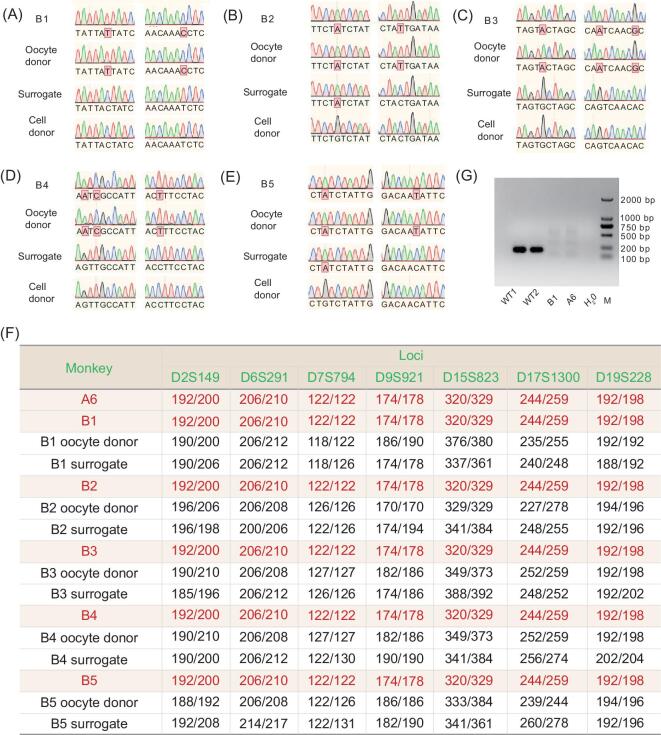
Analysis of the genetic origin and BMAL1 expression of cloned monkeys. (A–E) Examples of SNPs for B1–B5, respectively, showing mtDNA SNPs that were identical to those of the oocyte donor monkey, but different from those of surrogate monkey and donor fibroblasts. (F) Examples of STRs from ear tissue samples taken from five cloned monkeys (B1–B5), showing that their nuclear DNA was identical to that of the donor fibroblast, but different from those of their oocyte donor and surrogate monkeys. A more extensive list of the STRs is given in Table S2. (G) Reverse transcription PCR analysis of *BMAL1* expression on the blood sample from cloned monkey B1 showed a complete absence of the wild-type *BMAL1* transcript.

To confirm the genetic origin of the cloned monkeys, we analyzed single-nucleotide polymorphisms (SNPs) of mitochondrial DNA (mtDNA) and short-tandem repeats (STRs) of nuclear DNA [19,20]. For mtDNA, we found that the SNPs of the ND3 gene for the cloned monkeys were all identical to those of their respective oocyte-donor monkeys, but different from those of the surrogate monkeys and donor fibroblasts, consistent with the predominant contribution of donor oocyte mtDNA to the total mtDNA of SCNT embryos (Fig. 3A–E, Supplementary data S1). A total of 29 loci were used for STR analysis, and the results showed that all five cloned monkeys shared the same nuclear DNA of donor fibroblasts and the founder monkey A6, but that their nuclear DNA was different from those of the surrogate monkeys and oocyte donors (Fig. 3F, Supplementary Table S2).

Although *BMAL1*-specific editing was confirmed in the A6 founder monkey by whole-genome sequencing and PCR analysis, off-target analysis was also performed in the five cloned monkeys by PCR using genomic DNA from ear tissue. We found that no detectable mutation of potential off-target sites was predicted (Supplementary Table S3). We also detected no wild-type *BMAL1* transcripts in the blood of monkey B1, which was the only monkey old enough for blood collection (Fig. 3G).

## DISCUSSION

In this study, we have cloned five cynomolgus macaque monkeys by SCNT using fibroblasts obtained from a young adult male monkey in which BMAL1, a critical transcription factor for activating circadian rhythms, was knocked out by CRISPR/Cas9 gene editing. This founder monkey was shown to exhibit strong circadian disorder phenotypes. The genetic origin of the cloned monkeys was confirmed by the presence of the same *BMAL1* mutation genotypes and identical nuclear DNA in the cloned monkeys as those in the founder monkey, and the dominance of mtDNA by that of the donor oocyte. These results demonstrate that monkeys produced by CRISPR/Cas9 editing of a disease-related gene could be used for SCNT and to generate monkey clones with identical nuclear backgrounds, providing useful animal models of human diseases.

In our previous study [15], successful cloning of macaque monkeys with SCNT was performed by using female fetal fibroblasts from an aborted fetus as the somatic nucleus donor. In this study, we generated for the first time cloned monkeys using fibroblasts of a 16-month-old young adult male monkey, with success rates close to that obtained by SCNT of fetal fibroblasts. While the overall efficiency of SCNT remains low, it could be further improved by optimizing the epigenetic reprogramming conditions. The introduction of epigenetic modulators such as H3K9me3 demethylase Kdm4d was shown to facilitate the development of SCNT embryos [15,21–23]. Down-regulation of X chromosome genes caused by high-level *Xist* activation, as found in mouse and pig SCNT embryos [24–26], may also impair the development of cloned monkey embryos. Thus, impeding *Xist* expression is likely to improve the efficiency of monkey cloning. Besides, methods to correct other abnormal epigenetic reprogramming effects, such as aberrant DNA re-methylation [27] and the loss of H3K27me3 imprinting [25], are also likely to further improve efficiency.

Ideally, gene editing could be performed in cultured fibroblasts derived from a wild-type founder monkey and screened for fibroblast cell lines with precise gene targeting prior to SCNT. However, previous studies [28], confirmed by our preliminary studies, have shown that CRISPR/Cas9 editing of donor somatic cells greatly affects cell viability and leads to cell apoptosis. In the present study, we used fibroblasts from a founder monkey carrying a *BMAL1* gene mutation that was produced by CRISPR/Cas9 editing of intracytoplasmic sperm injection zygotes, circumventing the need for gene editing of fibroblasts. This founder monkey was chosen for its complete absence of BMAL1 expression and clear phenotypes resembling circadian disorders in humans (see accompanying paper). Despite complete BMAL1 ablation, CRISPR/Cas9 editing of *BMAL1* in the founder monkey had resulted in two genotypes of base-pair mutation (homogenous −8/−8 and heterogeneous −8/−8, +4,2PM) in different tissue cells. This genetic mosaicism was eliminated in our cloned monkeys, each of which carries only one of the two genotypes. Thus, the present cloning strategy is suitable for the cloning of gene-modified monkeys with clear phenotypes. Further characterization of the phenotypes of the five cloned monkeys will be performed when they reached appropriate ages.

## METHODS

### Animal ethics statement

The use and care of cynomolgus monkeys (*Macaca fascicularis*) complied with the guidelines of the Animal Advisory Committee at the Shanghai Institutes for Biological Science, Chinese Academy of Sciences, under the approved application entitled ‘Generation of gene-modified monkey models by somatic cell nuclear transfer' (ION-2018002). The monkeys used in this experiment were housed in an air-conditioned environment (temperature: 22 ± 1°C; humidity: 50% ± 5% RH) with a 12-h light/12-h dark cycle (lights-on time 07:00 to 19:00). All animals were fed a commercial monkey diet (Anmufei, Suzhou) twice daily, and with fruits and vegetables supplemented once daily.

### Fibroblast cell culture, and genotype and karyotype analysis

A small piece of sterilized skin tissue was obtained from the lateral thigh of the anesthetized *BMAL1*-edited founder monkey A6 (see accompanying paper). The tissue was washed three times in phosphate-buffered saline containing penicillin and streptomycin, cut into small pieces (1–2 mm^3^) and cultured as explants in a 6-cm culture dish. The fibroblast cells that migrated away from the tissue explants after 10 d were cultured in a 10-cm dish and passaged by one-third dilution every 3 d.

For *BMAL1* genotype analysis, the ear tissue, blood cells and cultured fibroblasts of the A6 monkey were collected for genome DNA extraction using a TIANamp Genomic DNA Kit (TIANGEN, DP304). The PCR products of the *BMAL1* target site specifically amplified by the primers (forward: 5′-ACCATCGGCTGCGTACACCTCTAT-3′; reverse: 5′-ATTTCAGGTGTGAGCCACTCCACC-3′) were cloned into a T vector and then sequenced for the genomic analysis.

For karyotype analysis, a confluent 10-cm dish of the A6 fibroblasts was incubated with 100 ng/ml colcemid for 4–6 h. The cells were then digested by 0.25% trypsin-ethylenediaminetetraacetic acid and treated with 0.075 M KCl at 37°C for 30 min. Hypotonic solution-treated cells were fixed in methanol and acetic acid (3:1 in volume) medium for 30 min and dropped onto pre-cleaned slides. Karyotypes were assessed by normal chromosome number counting.

### Super-ovulation and oocyte collection

Super-ovulation of female monkeys was performed as described previously [18]. Briefly, healthy female cynomolgus monkeys were intramuscularly injected with 25 IU recombinant human follitropin twice daily from day 3 of the menstrual cycle to day 11. On the night of day 11, 1000 IU of human chorionic gonadotrophin was intramuscularly injected, followed by oocyte collection from the follicles (2–8 mm in diameter) via laparoscopy and a negative-pressure suction system. The collected oocytes were cultured in pre-equilibrated hamster embryo culture medium 9 (HECM-9). Metaphase II-arrested oocytes were selected for SCNT.

### Monkey SCNT, embryo culturing and embryo transfer

The monkey SCNT procedure was the same as that previously reported [15]. Briefly, the spindle–chromosome complex was removed rapidly by a piezo-driven pipette under a spindle-imaging microscopic system (Oosight). The fibroblast was introduced to the perivitelline space through a slit in the zona pellucida that was created by laser irradiation and fused to the enucleated oocyte by virus (HVJ-E)-mediated fusion. The reconstructed monkey SCNT embryos were activated in TH3 (HEPES-buffered TALP medium, containing 0.3% bovine serum albumin) medium containing 5 mM ionomycin for 5 min and 2 mM 6-dimethylaminopurine for 5 h. The SCNT embryos were treated with 10 nM TSA for 10 h during and after activation, and injected with 10 pl of 1000 ng/ml *Kdm4d* mRNA at 6 h after activation.

The SCNT monkey embryos were cultured in HECM-9 medium at 37°C under 5% CO_2_. The embryos were transferred to HECM-9 medium supplemented with 5% fetal bovine serum after reaching the eight-cell stage, and the medium was changed every other day until the embryos reached the blastocyst stage. For embryo transfer, females with synchronous menstrual cycles whose ovaries had a stigma or fresh corpus luteum were used as surrogates. Embryos (at the two-to-eight-cell stage) were transferred to the oviduct.

### Genetic analysis of cloned monkeys

Genomic DNA extracted from ear tissue was used for short tandem repeats (STR) analysis. Locus-specific primers containing fluorescent dye (FAM/HEX/TMR) were used for PCR amplification. Fluorescent dye-labeled STR amplicons were diluted and mixed with internal size standard ROX500 and deionized formamide, and then capillary electrophoresed on an ABI PRISM 3730 genetic analyzer to obtain the raw data. The resultant raw data were analyzed with the program Gene Marker 2.2.0, which produces Excel documents including size and genotype information, DNA profiles and wave plots. For SNP analysis, mtDNA was also extracted from the monkey ear tissue samples. PCR with specific primers (forward: 5′-CCACTTCACATCAAACCATCACTT-3′; reverse: 5′-CAAGCAGCGAATACCAGCAAAA-3′) in mtDNA was performed with 35 cycles at 95°C for 30 s, 55°C for 30 s and 72°C for 1 min, followed by a 5-min extension step at 72°C. The PCR products were used for sequencing and the results were used for the SNP analysis.

### Reverse transcription PCR analysis

Total RNA was isolated with TRIzol^®^ Reagent (Invitrogen) from leucocytes from 0.5 ml blood from infant A6, infant B1 and two wild-type control monkeys, and reverse transcribed to cDNA using a PrimeScript™ RT reagent Kit with gDNA Eraser (Perfect Real Time, Takara, Japan) according to the manufacturer's instructions. Primers specific for *BMAL1* (forward; 5′-TAACCTCAGCTGCCTCGTTG-3′; reverse; 5′- TATTCATAACACGACGTGCC-3′) were used to amplify a 201-bp fragment from the wild *BMAL1* gene, but not for the mutant *BMAL1* gene. PCR was performed using a three-step amplification program of 40 cycles at 95°C for 10 s, 60°C for 15 s and 72°C for 30 s.

## Supplementary Material

Supplementary Files
