# Supplementary material for: Cloning of a gene-edited macaque monkey by somatic cell nuclear transfer
Source: Natl Sci Rev. 2019 Jan 24;6(1):101–8. doi: 10.1093/nsr/nwz003 (PMC8291622; doi:10.1093/nsr/nwz003)
Supplement: Supplementary Files [file nwz003_supplemental_files.zip › Supplementary Data S1.MtDNA sequence of the ND3 gene.docx]

**Supplementary Data S1. MtDNA sequence of the *ND3* gene**

**B1:**

GATGTTATCTGACTACTCCTCTACATTTCTATCTATTGATGAGGATCCTACTCTTTTAGTATAACAAGTACAATTGACTTCCAATCAATCAGTTTTGACAACATTCAAAAAAGAGTAATTAACCTAGTGCTAGCCTTAACAGTCAACACCCTATTAACCTCGCTACTGATAATTATCATATTCTGGTTACCTCAACTCAACTCCTACGCAGAAAAAACTAGCCCCTACGAATGTGGATTTGATCCCCTAAACCCCGCTCGCATCCCATTCTCAATAAAATTCTTCCTAGTTGCCATTACCTTCCTACTATTTGACCTAGAGATCGCCCTATTATTATCCTTACCATGAGCCATTCAAACAACAAACCTCCCAACAATAATCAAATCAACCATCGCCTTTATTATTATCCTAATTCTCAGCCTAGCCTATGAATGAACTCAAAAGGGGCTAGACTGAGCCGAATTGGTAAGTAGTTTAAACAAAATAAATGATTTCGACTCATTAGATTATGATAACCATACTAACCAAATGACCCCCACCTATATAAATATCATACTAGCATTTACTATCTCTCTCCTAGGCATACTAACCTACCGCTCACACTTAGTAGCTTCTCTCCTCTGCCTAGAAGGAATAATAATATCACTCTTTATCATAACCACTCTTATTGCCTCAAACATACACTTCCCCCTAATCAACA

**Oocyte donor of B1 (573#):**

GATGTTATCTGACTACTCCTCTACATTTCTATCTATTGATGAGGATCCTACTCTTTTAGTATAACAAGTACAATTGACTTCCAATCAATCAGTTTTGACAACATTCAAAAAAGAGTAATTAACCTAGTGCTAGCCTTAACAGTCAACACCCTATTAACCTCGCTACTGATAATTATCATATTCTGGTTACCTCAACTCAACTCCTACGCAGAAAAAACTAGCCCCTACGAATGTGGATTTGATCCCCTAAACCCCGCTCGCATCCCATTCTCAATAAAATTCTTCCTAGTTGCCATTACCTTCCTACTATTTGACCTAGAGATCGCCCTATTATTATCCTTACCATGAGCCATTCAAACAACAAACCTCCCAACAATAATCAAATCAACCATCGCCTTTATTATTATCCTAATTCTCAGCCTAGCCTATGAATGAACTCAAAAGGGGCTAGACTGAGCCGAATTGGTAAGTAGTTTAAACAAAATAAATGATTTCGACTCATTAGATTATGATAACCATACTAACCAAATGACCCCCACCTATATAAATATCATACTAGCATTTACTATCTCTCTCCTAGGCATACTAACCTACCGCTCACACTTAGTAGCTTCTCTCCTCTGCCTAGAAGGAATAATAATATCACTCTTTATCATAACCACTCTTATTGCCTCAAACATACACTTCCCCCTAATCAACA

**Surrogate of B1 (377#):**

GATGTTATCTGACTACTCCTCTACATTTCTATCTATTGATGAGGATCCTACTCTTTTAGTATAACAAGTACAATTGACTTCCAATCAATCAGTTTTGACAGCATTCAAAAAAGAGTAATTAACCTAGTGCTAGCCTTAACAGTCAACACCCTATTAACCTCGCTATTGATAATTATCATATTCTGATTACCTCAACTCAACTCCTACGCAGAAAAAACTAGCCCCTACGAATGTGGATTTGATCCCCTAAACCCCGCTCGCATCCCATTCTCAATAAAATTCTTCCTAGTTGCCATTACCTTCCTACTATTTGACCTAGAGATCGCCCTATTACTATCCTTACCATGAGCCATTCAAACAACAAATCTCCCAACAATAATCAAATCAACCATCGCCTTTATTATTATCCTAATTCTCAGCCTAGCCTATGAATGAACTCAAAAGGGGCTAGACTGAGCCGAATTGGTAAGTAGTTTAAACAAAATAAATGATTTCGACTCATTAGATTATGATAACCATACTAACCAAATGACCCCCACCTATATAAACATCATACTAGCATTTACTATCTCTCTCCTAGGCATACTAACCTACCGCTCACACTTAGTAGCTTCTCTCCTCTGCCTAGAAGGAATAATAATATCACTCTTTATCATAACCACTCTTATCGCCTCAAACATACACTTCCCCCTAATCAACATTA

**B2:**

GATGTTATCTGACTACTCCTCTACATTTCTATCTATTGATGAGGATCCTACTCTTTTAGTATAACAAGTACAATTGACTTCCAATCAATCAGTTTTGACAGCATTCAAAAAAGAGTAATTAACCTAGTGCTAGCCTTAACAGTCAACACCCTATTAACCTCGCTATTGATAATTATCATATTCTGGTTACCTCAACTCAACTCCTACGCAGAAAAAACTAGCCCCTACGAATGTGGATTTGATCCCCTAAACCCCGCTCGCATCCCATTCTCAATAAAATTCTTCCTAGTTGCCATTACCTTCCTACTATTTGACCTAGAGATCGCCCTATTACTATCCTTACCATGAGCCATTCAAACAACAAATCTCCCAACAATAATCAAATCAACCATCGCCTTTATTATTATCCTAATTCTCAGCCTAGCCTATGAATGAACTCAAAAGGGGCTAGACTGAGCCGAATTGGTAAGTAGTTTAAACAAAATAAATGATTTCGACTCATTAGATTATGATAACCATACTAACCAAATGACCCCCACCTATATAAACATCATACTAGCATTTACTATCTCTCTCCTAGGCATACTAACCTACCGCTCACACTTAGTAGCTTCTCTCCTCTGCCTAGAAGGAATAATAATATCACTCTTTATCATAACCACTCTTATCGCCTCAAACATACACTTCCCCCTAATCAAC

**Oocyte donor of B2 (454#):**

GATGTTATCTGACTACTCCTCTACATTTCTATCTATTGATGAGGATCCTACTCTTTTAGTATAACAAGTACAATTGACTTCCAATCAATCAGTTTTGACAGCATTCAAAAAAGAGTAATTAACCTAGTGCTAGCCTTAACAGTCAACACCCTATTAACCTCGCTATTGATAATTATCATATTCTGGTTACCTCAACTCAACTCCTACGCAGAAAAAACTAGCCCCTACGAATGTGGATTTGATCCCCTAAACCCCGCTCGCATCCCATTCTCAATAAAATTCTTCCTAGTTGCCATTACCTTCCTACTATTTGACCTAGAGATCGCCCTATTACTATCCTTACCATGAGCCATTCAAACAACAAATCTCCCAACAATAATCAAATCAACCATCGCCTTTATTATTATCCTAATTCTCAGCCTAGCCTATGAATGAACTCAAAAGGGGCTAGACTGAGCCGAATTGGTAAGTAGTTTAAACAAAATAAATGATTTCGACTCATTAGATTATGATAACCATACTAACCAAATGACCCCCACCTATATAAACATCATACTAGCATTTACTATCTCTCTCCTAGGCATACTAACCTACCGCTCACACTTAGTAGCTTCTCTCCTCTGCCTAGAAGGAATAATAATATCACTCTTTATCATAACCACTCTTATCGCCTCAAACATACACTTCCCCCTAATCAAC

**Surrogate of B2 (567#):**

GATGTTATCTGACTACTCCTCTACATTTCTATCTATTGATGAGGATCCTACTCTTTTAGTATAACAAGTACAATTGACTTCCAATCAATCAGTTTTGACAATATTCAAAAAAGAGTAATTAACCTAGTGCTAGCCTTAACAGTCAACACCCTATTAACCTCGCTACTGATAATTATCATATTCTGGTTACCTCAACTCAACTCCTACGCAGAAAAAACTAGCCCCTACGAATGTGGATTTGATCCCCTAAACCCCGCTCGCATCCCATTCTCAATAAAATTCTTCCTAGTTGCCATTACCTTCCTACTATTTGACCTAGAGATCGCCCTATTACTATCCTTACCATGAGCCATTCAAACAACAAATCTCCCAACAATAATCAAATCAACCATCGCCTTTATTATTATCCTAATTCTCAGCCTAGCCTATGAATGAACTCAAAAGGGGCTAGACTGAGCCGAATTGGTAAGTAGTTTAAACAAAATAAATGATTTCGACTCATTAGATTATGATAACCATACTAACCAAATGACCCCCACCTATATAAACATCATACTAGCATTTACTATCTCTCTCCTAGGCATACTAACCTACCGCTCACACTTGGTAGCTTCTCTCCTCTGCCTAGAAGGAATAATAATATCACTCTTTATCATAACCACTCTTATCGCCTCAAACATACACTTCCCCCTAATCAACA

**B3:**

GATGTTATCTGACTACTCCTCTACATTTCTATTTATTGATGAGGATCCTACTCTTTTAGTATAACAAGTACAATTGACTTCCAATCAATCAGTTTTGACAACATTCAAAAAAGAGTAATTAACCTAGTACTAGCCTTAACAATCAACGCCCTATTAACCTTGCTACTGATAATTATCATATTCTGGTTACCCCAACTCAACTCCTACGCAGAAAAAACTAGCCCCTACGAATGTGGATTTGACCCCCTAAACCCTGCCCGCATCCCATTCTCAATAAAATTCTTCCTAATCGCCATTACTTTCCTACTATTTGACATAGAAATCGCCCTACTACTATCCTTACCATGAGCCATTCAAACAACAGACCTCCCAACAATAATCAAATCGACCTTCGCTTTCATTATTATCCTAATTCTCAGCCTAGCCTATGAATGAACTCAAAAGGGGCTAGACTGAGCTGAATTGGTAAGTAGTTTAAACAAAATAAATGATTTCGACTCATTAGATTATGATAACCATACTAACCAAATGACCCCCACCTATATAAACATCATACTAGCATTTACTATCTCTCTTCTAGGCATACTAACCTACCGCTCACACTTAGTAGCTTCTCTCCTCTGCCTAGAAGGAATAATAATATCACTCTTTATCATAGCCACTCTTATTGCCTCAAACACACACTTCCCCCTAGTCAACA

**Oocyte donor of B3 (534#):**

GATGTTATCTGACTACTCCTCTACATTTCTATTTATTGATGAGGATCCTACTCTTTTAGTATAACAAGTACAATTGACTTCCAATCAATCAGTTTTGACAACATTCAAAAAAGAGTAATTAACCTAGTACTAGCCTTAACAATCAACGCCCTATTAACCTTGCTACTGATAATTATCATATTCTGGTTACCCCAACTCAACTCCTACGCAGAAAAAACTAGCCCCTACGAATGTGGATTTGACCCCCTAAACCCTGCCCGCATCCCATTCTCAATAAAATTCTTCCTAATCGCCATTACTTTCCTACTATTTGACATAGAAATCGCCCTACTACTATCCTTACCATGAGCCATTCAAACAACAGACCTCCCAACAATAATCAAATCGACCTTCGCTTTCATTATTATCCTAATTCTCAGCCTAGCCTATGAATGAACTCAAAAGGGGCTAGACTGAGCTGAATTGGTAAGTAGTTTAAACAAAATAAATGATTTCGACTCATTAGATTATGATAACCATACTAACCAAATGACCCCCACCTATATAAACATCATACTAGCATTTACTATCTCTCTTCTAGGCATACTAACCTACCGCTCACACTTAGTAGCTTCTCTCCTCTGCCTAGAAGGAATAATAATATCACTCTTTATCATAGCCACTCTTATTGCCTCAAACACACACTTCCCCCTAGTCAACA

**Surrogate of B3 (511#):**

GATGTTATCTGACTACTCCTCTACATTTCTATCTATTGATGAGGATCCTACTCTTTTAGTATAACAAGTACAATTGACTTCCAATCAATCAGTTTTGACAACATTCAAAAAAGAGTAATTAACCTAGTGCTAGCCTTAACAGTCAACACCCTATTAACCTCGCTACTGATAATTATCATATTCTGGTTACCCCAACTCAACTCCTACGCAGAAAAAACTAGCCCCTACGAATGTGGATTTGATCCCCTAAACCCCGCTCGCATCCCATTCTCAATAAAATTCTTCCTAGTTGCCATTACCTTCCTACTATTTGACCTAGAGATCGCCCTATTACTATCCTTACCATGAGCCATTCAAACAACAAATCTCCCAACAATAATCAAATCAACCATCGCCTTTATTATTATCCTAATTCTCAGCCTAGCCTATGAATGAACTCAAAAGGGGCTAGACTGAGCCGAATTGGTAAGTAGTTTAAACAAAATAAATGATTTCGACTCATTAGATTATGATAACCATACTAACCAAATGACCCCCACCTATATAAACATCATACTAGCATTTACTATCTCTCTCCTAGGCATACTAACCTACCGCTCACACTTGGTAGCTTCTCTCCTCTGCCTAGAAGGAATAATAATATCACTCTTTATCATAACCACTCTTATCGCCTCAAACATACACTTCCCCCTAATCAACA

**B4:**

GATGTTATCTGACTACTCCTCTACATTTCTATTTATTGATGAGGATCCTACTCTTTTAGTATAACAAGTACAATTGACTTCCAATCAATCAGTTTTGACAACATTCAAAAAAGAGTAATTAACCTAGTACTAGCCTTAACAATCAACGCCCTATTAACCTTGCTACTGATAATTATCATATTCTGGTTACCCCAACTCAACTCCTACGCAGAAAAAACTAGCCCCTACGAATGTGGATTTGACCCCCTAAACCCTGCCCGCATCCCATTCTCAATAAAATTCTTCCTAATCGCCATTACTTTCCTACTATTTGACATAGAAATCGCCCTACTACTATCCTTACCATGAGCCATTCAAACAACAGACCTCCCAACAATAATCAAATCGACCTTCGCTTTCATTATTATCCTAATTCTCAGCCTAGCCTATGAATGAACTCAAAAGGGGCTAGACTGAGCTGAATTGGTAAGTAGTTTAAACAAAATAAATGATTTCGACTCATTAGATTATGATAACCATACTAACCAAATGACCCCCACCTATATAAACATCATACTAGCATTTACTATCTCTCTTCTAGGCATACTAACCTACCGCTCACACTTAGTAGCTTCTCTCCTCTGCCTAGAAGGAATAATAATATCACTCTTTATCATAGCCACTCTTATTGCCTCAAACACACACTTCCCCCTAGTCAACA

**Oocyte donor of B4 (534#):**

GATGTTATCTGACTACTCCTCTACATTTCTATTTATTGATGAGGATCCTACTCTTTTAGTATAACAAGTACAATTGACTTCCAATCAATCAGTTTTGACAACATTCAAAAAAGAGTAATTAACCTAGTACTAGCCTTAACAATCAACGCCCTATTAACCTTGCTACTGATAATTATCATATTCTGGTTACCCCAACTCAACTCCTACGCAGAAAAAACTAGCCCCTACGAATGTGGATTTGACCCCCTAAACCCTGCCCGCATCCCATTCTCAATAAAATTCTTCCTAATCGCCATTACTTTCCTACTATTTGACATAGAAATCGCCCTACTACTATCCTTACCATGAGCCATTCAAACAACAGACCTCCCAACAATAATCAAATCGACCTTCGCTTTCATTATTATCCTAATTCTCAGCCTAGCCTATGAATGAACTCAAAAGGGGCTAGACTGAGCTGAATTGGTAAGTAGTTTAAACAAAATAAATGATTTCGACTCATTAGATTATGATAACCATACTAACCAAATGACCCCCACCTATATAAACATCATACTAGCATTTACTATCTCTCTTCTAGGCATACTAACCTACCGCTCACACTTAGTAGCTTCTCTCCTCTGCCTAGAAGGAATAATAATATCACTCTTTATCATAGCCACTCTTATTGCCTCAAACACACACTTCCCCCTAGTCAACA

**Surrogate of B4 (419#):**

GATGTTATCTGACTACTCCTCTACATTTCTATCTATTGATGAGGATCCTACTCTTTTAGTATAACAAGTACAATTGACTTCCAATCAATCAGTTTTGACAGCATTCAAAAAAGAGTAATTAACCTAGTGCTAGCCTTAACAGTCAACACCCTATTAACCTCGCTATTGATAATTATCATATTCTGGTTACCTCAACTCAACTCCTACGCAGAAAAAACTAGCCCCTACGAATGTGGATTTGATCCCCTAAACCCCGCTCGCATCCCATTCTCAATAAAATTCTTCCTAGTTGCCATTACCTTCCTACTATTTGACCTAGAGATCGCCCTATTACTATCCTTACCATGAGCCATTCAAACAACAAATCTCCCAACAATAATCAAATCAACCATCGCCTTTATTATTATCCTAATTCTCAGCCTAGCCTATGAATGAACTCAAAAGGGGCTAGACTGAGCCGAATTGGTAAGTAGTTTAAACAAAATAAATGATTTCGACTCATTAGATTATGATAACCATACTAACCAAATGACCCCCACCTATATAAACATCATACTAGCATTTACTATCTCTCTCCTAGGCATACTAACCTACCGCTCACACTTAGTAGCTTCTCTCCTCTGCCTAGAAGGAATAATAATATCACTCTTTATCATAACCACTCTTATCGCCTCAAACATACACTTCCCCCTAATCAAC

**B5:**

GATGTTATCTGACTACTCCTCTACATTTCTATCTATTGATGAGGATCCTACTCTTTTAGTATAACAAGTACAATTGACTTCCAATCAATCAGTTTTGACAATATTCAAAAAAGAGTAATTAACCTAGTGCTAGCCTTAACAGTCAACACCCTATTAACCTCGCTACTGATAATTATCATATTCTGGTTACCTCAACTCAACTCCTACGCAGAAAAAACTAGCCCCTACGAATGTGGATTTGATCCCCTAAACCCCGCTCGCATCCCATTCTCAATAAAATTCTTCCTAGTTGCCATTACCTTCCTACTATTTGACCTAGAGATCGCCCTATTACTATCCTTACCATGAGCCATTCAAACAACAAATCTCCCAACAATAATCAAATCAACCATCGCCTTTATTATTATCCTAATTCTCAGCCTAGCCTATGAATGAACTCAAAAGGGGCTAGACTGAGCCGAATTGGTAAGTAGTTTAAACAAAATAAATGATTTCGACTCATTAGATTATGATAACCATACTAACCAAATGACCCCCACCTATATAAACATCATACTAGCATTTACTATCTCTCTCCTAGGCATACTAACCTACCGCTCACACTTGGTAGCTTCTCTCCTCTGCCTAGAAGGAATAATAATATCACTCTTTATCATAACCACTCTTATCGCCTCAAACATACACTTCCCCCTAATCAACA

**Oocyte donor of B5 (546#):**

GATGTTATCTGACTACTCCTCTACATTTCTATCTATTGATGAGGATCCTACTCTTTTAGTATAACAAGTACAATTGACTTCCAATCAATCAGTTTTGACAATATTCAAAAAAGAGTAATTAACCTAGTGCTAGCCTTAACAGTCAACACCCTATTAACCTCGCTACTGATAATTATCATATTCTGGTTACCTCAACTCAACTCCTACGCAGAAAAAACTAGCCCCTACGAATGTGGATTTGATCCCCTAAACCCCGCTCGCATCCCATTCTCAATAAAATTCTTCCTAGTTGCCATTACCTTCCTACTATTTGACCTAGAGATCGCCCTATTACTATCCTTACCATGAGCCATTCAAACAACAAATCTCCCAACAATAATCAAATCAACCATCGCCTTTATTATTATCCTAATTCTCAGCCTAGCCTATGAATGAACTCAAAAGGGGCTAGACTGAGCCGAATTGGTAAGTAGTTTAAACAAAATAAATGATTTCGACTCATTAGATTATGATAACCATACTAACCAAATGACCCCCACCTATATAAACATCATACTAGCATTTACTATCTCTCTCCTAGGCATACTAACCTACCGCTCACACTTGGTAGCTTCTCTCCTCTGCCTAGAAGGAATAATAATATCACTCTTTATCATAACCACTCTTATCGCCTCAAACATACACTTCCCCCTAATCAACA

**Surrogate of B5 (457#):**

GATGTTATCTGACTACTCCTCTACATTTCTATCTATTGATGAGGATCCTACTCTTTTAGTATAACAAGTACAATTGACTTCCAATCAATCAGTTTTGACAACATTCAAAAAAGAGTAATTAACCTAGTGCTAGCCTTAACAGTCAACACCCTATTAACCTCACTACTGATAATTATCATATTCTGGTTACCTCAACTCAACTCCTACGCAGAAAAAACTAGCCCCTACGAATGTGGATTTGATCCCCTAAATCCCGCTCGCATCCCATTCTCAATAAAATTTTTCCTAGTTGCCATTACCTTCCTACTATTTGACCTAGAGATCGCCCTATTACTATCCTTACCATGAGCCATTCAAACAACAAACCTCCCAACAATAATCAAATCAACCATCGCCTTTATTATTATCCTAATTCTCAGCCTAGCCTATGAATGAACTCAAAAGGGGCTAGACTGAGCCGAATTGGTAAGTAGTTTAAACAAAATAAATGATTTCGACTCATTAGATTATGATAACCATACTAACCAAATGACCCCCACCTATATAAACATCATACTAGCATTTACTATCTCTCTCCTAGGCATACTAACCTACCGCTCACACTTAGTAGCTTCTCTCCTCTGCCTAGAAGGAATAATAATATCACTCTTTATCATAACCACTCTTATTGCCTCAAACATACACTTCCCCCTAATCAACA

**Cell donor (A6):**

GATGTTATCTGACTACTCCTCTACATTTCTGTCTATTGATGAGGATCCTACTCTTTTAGTATAACAAGTACAATTGACTTCCAATCAATCAGTTTTGACAACATTCAAAAAAGAGTAATTAACCTAGTGCTAGCCTTAACAGTCAACACCCTATTAACCTCGCTACTGATAATTATCATATTCTGGTTACCTCAACTCAACTCCTACGCAGAAAAAACTAGCCCCTACGAATGTGGATTTGATCCCCTAAACCCCGCTCGCATCCCATTCTCAATAAAATTCTTCCTAGTTGCCATTACCTTCCTACTATTTGACCTAGAGATCGCCCTATTACTATCCTTACCATGAGCCATTCAAACAACAAATCTCCCAACAATAATCAAATCAACCATCGCCTTTATTATTATCCTAATTCTCAGCCTAGCCTATGAATGAACTCAAAAGGGGCTAGACTGAGCCGAATTGGTAAGTAGTTTAAACAAAATAAATGATTTCGACTCATTAGATTATGATAACCATACTAACCAAATGACCCCCACCTATATAAACATCATACTAGCATTTACTATCTCTCTCCTAGGCATACTAACCTACCGCTCACACTTGGTAGCTTCTCTCCTCTGCCTAGAAGGAATAATAATATCACTCTTTATCATAACCACTCTTATCGCCTCAAACATACACTTCCCCCTAATAAACA
