# Supplementary material for: Cloning of a gene-edited macaque monkey by somatic cell nuclear transfer
Source: Natl Sci Rev. 2019 Jan 24;6(1):101–8. doi: 10.1093/nsr/nwz003 (PMC8291622; doi:10.1093/nsr/nwz003)
Supplement: Supplementary Files [file nwz003_supplemental_files.zip › Supplementary Table S1. Information of oocyte donors and embryo recipients that produced live births.docx]

| # of oocyte donor (age) | # of Surrogate  (age) | # of embryos transferred | # of GS | # of fetus | Outcome |
| --- | --- | --- | --- | --- | --- |
| 573# (6.5 year) | 377# (7.5 year) | 5 | 2 | 1 | Caesarean section at D145 (B1) |
| 454# (9.5 year) | 567# (6.5 year) | 6 | 1 | 1 | Caesarean section at D151 (B2) |
| 534# (6.5 year) | 511# (6.5 year) | 5 | 1 | 1 | Caesarean section at D150 (B3) |
| 534# (6.5 year) | 419# (8 year) | 7 | 2 | 1 | Caesarean section at D155 (B4) |
| 546# (6.5 year) | 457# (8 year) | 5 | 1 | 1 | Caesarean section at D155 (B5) |

**Supplementary Table S1. Information of oocyte donors and embryo recipients that produced live births.**
