# Supplementary material for: Cloning of a gene-edited macaque monkey by somatic cell nuclear transfer
Source: Natl Sci Rev. 2019 Jan 24;6(1):101–8. doi: 10.1093/nsr/nwz003 (PMC8291622; doi:10.1093/nsr/nwz003)
Supplement: Supplementary Files [file nwz003_supplemental_files.zip › Supplementary Table S3.Off target analysis of the cloned monkeys.docx]

| **Sites** | **Target sequence** | **Chr** | **Gene** | **Mismatches** | **A6** | **B1** | **B2** | **B3** | **B4** | **B5** |
| --- | --- | --- | --- | --- | --- | --- | --- | --- | --- | --- |
| **SgRNA-1** | **GCAGTCGTCCAATTGCAACGAGG** | **14** | **ARNTL** | **0** |  |  |  |  |  |  |
| **OT1** | **GgAGTCtcCCAATTGCcACGAGG** | **7** | **ARNT2** | **4** | **-** | **-** | **-** | **-** | **-** | **-** |
| **OT2** | **cCAGTtGTCCAcTTGCAgCGTGG** | **7** | **NRXN3** | **4** | **-** | **-** | **-** | **-** | **-** | **-** |
| **OT3** | **GCAcTCtTCCcATTGCAcCGTGG** | **X** | **None** | **4** | **-** | **-** | **-** | **-** | **-** | **-** |
| **OT4** | **GCtGTCcTCCAATTGaAcCGTGG** | **11** | **PIK3C2G** | **4** | **-** | **-** | **-** | **-** | **-** | **-** |
| **OT5** | **GCAGgCtTCatATTGCAACGTGG** | **11** | **None** | **4** | **-** | **-** | **-** | **-** | **-** | **-** |
| **OT6** | **GCAGgCaTtCAATgGCAACGGGG** | **1** | **None** | **4** | **-** | **-** | **-** | **-** | **-** | **-** |
| **OT7** | **GgAGTCaTCaAATTGCAAaGTGG** | **16** | **YPEL2** | **4** | **-** | **-** | **-** | **-** | **-** | **-** |
|  | | | | | | | | | | |
| **SgRNA-3** | **GTTTCTCGGCACGCGATAGATGG** | **14** | **ARNTL** | **0** |  |  |  |  |  |  |
| **OT9** | **GTTTCTCGGCAtGCGgTtGAGGG** | **15** | **None** | **3** | **-** | **-** | **-** | **-** | **-** | **-** |
| **OT10** | **GTTTCTCtGCAtaCGATgGAGGG** | **4** | **None** | **4** | **-** | **-** | **-** | **-** | **-** | **-** |
| **OT11** | **cTTTCTCaGCACGtGAaAGATGG** | **8** | **None** | **4** | **-** | **-** | **-** | **-** | **-** | **-** |
| **OT12** | **GTTTCTaGGtACctGATAGAAGG** | **10** | **None** | **4** | **-** | **-** | **-** | **-** | **-** | **-** |
| **OT13** | **GTTTCTCGGacaGaGATAGAAGG** | **15** | **None** | **4** | **-** | **-** | **-** | **-** | **-** | **-** |
| **OT14** | **GTTTCTaGGtAaGaGATAGATGG** | **1** | **None** | **4** | **-** | **-** | **-** | **-** | **-** | **-** |
| **OT15** | **GTcTCTCGGCtCtgGATAGAGGG** | **16** | **None** | **4** | **-** | **-** | **-** | **-** | **-** | **-** |

**Supplementary Table S3. Off target analysis of the cloned monkeys**
