# Supplementary material for: Cloning of a gene-edited macaque monkey by somatic cell nuclear transfer
Source: Natl Sci Rev. 2019 Jan 24;6(1):101–8. doi: 10.1093/nsr/nwz003 (PMC8291622; doi:10.1093/nsr/nwz003)
Supplement: Supplementary Files [file nwz003_supplemental_files.zip › Table 1. Statistics on the development of SCNT embryos.docx]

| **Cell passage** | **Embryos transferred** | **Surrogates** | **Pregnancies** | **Live birth (No.)** |
| --- | --- | --- | --- | --- |
| **2** | **118** | **23** | **7** | **1 (B1)** |
| **3** | **148** | **30** | **4** | **1 (B2)** |
| **4** | **59** | **12** | **5** | **3 (B3, B4, B5)** |
| **Total** | **325** | **65** | **16** | **5** |

**Table 1. Statistics on the development of SCNT embryos**
