# Supplementary material for: Cloning of a gene-edited macaque monkey by somatic cell nuclear transfer
Source: Natl Sci Rev. 2019 Jan 24;6(1):101–8. doi: 10.1093/nsr/nwz003 (PMC8291622; doi:10.1093/nsr/nwz003)
Supplement: Supplementary Files [file nwz003_supplemental_files.zip › NSR_MS-2018-234.R2.doc]

**Table 1. Statistics on the Development of SCNT Embryos**

**Supplementary Data S1. MtDNA sequence of the ND3 gene**

**Supplementary Table S1. Information of oocyte donors and embryo recipients that produced live births**

**Supplementary Table S2. STR analysis of the cloned monkeys**

**Supplementary Table S3. Off target analysis of the cloned monkeys**
